# Anti-proliferative activity and cell cycle arrest induced by evodiamine on paclitaxel-sensitive and -resistant human ovarian cancer cells

**DOI:** 10.1038/srep16415

**Published:** 2015-11-10

**Authors:** Zhang-Feng Zhong, Wen Tan, Sheng-Peng Wang, Wen-An Qiang, Yi-Tao Wang

**Affiliations:** 1 University of Macau, Institute of Chinese Medical Sciences, State Key Laboratory of Quality Research in Chinese Medicine, Macau, China; 2 Division of Reproductive Science in Medicine, Department of Obstetrics and Gynecology, Feinberg School of Medicine at Northwestern University, Chicago, Illinois, United States of America; 3 School of Pharmacy, Lanzhou University, Lanzhou, Gansu, China

## Abstract

Chemo-resistance is the main factor for poor prognosis in human ovarian epithelial cancer. Active constituents derived from Chinese medicine with anti-cancer potential might circumvent this obstacle. In our present study, evodiamine (EVO) derived from Evodia rutaecarpa (Juss.) Benth suppressed the proliferation of human epithelial ovarian cancer, A2780 and the related paclitaxel-resistant cell lines and did not cause cytotoxicity, as confirmed by the significant decline of clone formation and the representative alterations of CFDA-SE fluorescence. Meanwhile, EVO induced cell cycle arrest in a dose- and time-dependent manner. This disturbance might be mediated by the cooperation of Cyclin B1 and Cdc2, including the up-regulation of Cyclin B1, p27, and p21, and activation failure of Cdc2 and pRb. MAPK signaling pathway regulation also assisted in this process. Furthermore, chemo-sensitivity potential was enhanced as indicated in A2780/PTX^R^ cells by the down-regulation of MDR-1 expression, accompanied by MDR-1 function suppression. Taken together, we confirmed initially that EVO exerted an anti-proliferative effect on human epithelial ovarian cancer cells, A2780/WT and A2780/PTX^R^, induced G2/M phase cell cycle arrest, and improved chemo-resistance. Overall, we found that EVO significantly suppressed malignant proliferation in human epithelial ovarian cancer, thus proving to be a potential anti-cancer agent in the future.

Chemo-resistance is the main factor for poor prognosis in human ovarian epithelial cancer. As a result, since the year 2000 when X-linked inhibitors for apoptotic proteins (Xiap) assisted by p53 status were regarded as important targets for chemo-resistance in human ovarian epithelial cancer^1^, extensive investigations have concentrated on Xiap and the PI3K/Akt pathway^2^,^3^, BRCA1/2 alterations^4^, epithelial mesenchymal transition and cancerous ovarian stem cells^5^,^6^, and even considered metabolic alterations and epigenetic therapies^7^,^8^, in order to circumvent chemo-resistance.

Cell division cycle protein 2 (Cdc2) or cyclin-dependent kinase 1 (Cdk1) leads the entry into M phase and is also a key regulator in cell cycle progression by binding to cyclin kinases and causing phosphorylation. Over-expressed Cdc2 phosphorylating survivin has been found to be one of the causes of paclitaxel-resistant ovarian cancer^9^. For example, a reduction of Cdc2 was induced by down-regulation of BRCA1, which conferred paclitaxel resistance in breast cancer cells^10^. An inappropriate activation of Cdc2 induced by cyclin A1 contributed to an apoptotic and mitotic catastrophe in ovarian cancer^11^. Cdc2 siRNA also increased the sensitivity to cisplatin-induced apoptosis in ovarian cancer cells^12^. The phosphorylation inhibition of Cdc2 at Tyr 15 mediated DNA damage in UHRF1 (ubiquitin-like PHD and RING finger domain-containing 1) depletion ovarian cancer cells^13^.

Cyclin B1 is another important regulatory protein in the cell cycle, and it interacts with Cdc2 to form the cyclin B1-Cdk1 complex, promoting mitotic initiation^14^. These proteins prefer to over-express in low-malignant-potential tumors rather than epithelial ovarian cancer to develop tumorigenesis^15^. Cyclin binding and phosphorylation activation at Thr161 of Cdc2 are both required for Cdc2 activation for mitosis in cell cycle progression. p27 is a cyclin-dependent kinase inhibitor which binds to Cdc2 to prevent cell cycle transition. p27 and p21 are regarded as potential tumor suppressors, and low levels of p27 and high levels of phospho-Rb were found to significantly correlate with poor patient survival in ovarian cancer^16^. Rb is another signature for human high-grade serous epithelial ovarian cancer^17^, and it is phosphorylated by cyclin D kinases to lead to progression into the S phase of the cell cycle^18^. ERK and p38 are the main factors in the MAPK pathway, which also regulates cell cycle distribution in ovarian cancer. The Ras pathway is mutated in low-grade serous ovarian carcinomas^19^ where resistant cells are characterized by activation of the Ras/ERK pathway^20^. P-glycoprotein (P-gp) expression and function are also clinically significant in patients with ovarian cancer therapy^21^.

Evodiamine (EVO) is an indole alkaloid derived from Chinese medicine, Evodia rutaecarpa (Juss.) Benth. Reports show that it exerts anti-cancer potential in a wide range of cancer cell lines, including non-small-cell cancer cells, osteosarcoma cells, lung cancer cells, glioblastoma cells, etc. The mechanisms involved were found to suppress malignant proliferation, induce cell cycle arrest, and inhibit invasion and metastasis^22^,^23^,^24^,^25^,^26^,^27^,^28^,^29^,^30^,^31^,^32^,^33^,^34^,^35^,^36^,^37^,^38^,^39^,^40^,^41^,^42^,^43^,^44^. However, there is no report on human epithelial ovarian cancer cells and the related chemo-resistant cancer cells. Therefore, our present study was designed to investigate the effect of EVO on chemo-sensitive and -resistant human epithelial ovarian cancer and the underlying mechanisms.

## Materials and Methods

### Reagents

EVO, paclitaxel (PTX), crystal violet, and 3-[4,5-dimethyl-2-thiazolyl]-2,5-diphenyl tetrazolium bromide (MTT) were purchased from Sigma-Aldrich (St. Louis, MO). Dulbecco’s Modified Eagle Medium (DMEM), fetal bovine serum (FBS), penicillin (100 U/ml)-streptomycin (100 μg/ml), phosphate-buffered saline (PBS) and 0.25% w/v trypsin/1 mM EDTA from Gibco Life Technologies (Grand Island, USA) were used for cell culture. Calcium AM and carboxyfluorescein diacetate succinimidyl ester (CFDA-SE) were purchased from Molecular Probes (Grand Island, USA). The lactate dehydrogenase (LDH) release detection kit was purchased from Roche Diagnostics (Mannheim, Germany). The primary and secondary antibodies against Cyclin B1, p27, p21, Rb, p-Cdc2 (Thr161), Cdc2, p-ERK (Thr202/Tyr204), ERK, p38, Ras, GAPDH, and β-actin were purchased from Cell Signaling Technology (Danvers, MA). MDR-1 was obtained from Santa Cruz (Santa Cruz, USA).

### Cell lines and Cell culture

Human ovarian epithelial cancer cells, A2780/WT were used for anti-proliferation study. The paclitaxel-resistant A2780 cells were established by stepwise exposure to increased concentrations of paclitaxel, as previously described^45^. Cells were cultured in RPMI 1640 medium with penicillin (100 U/ml)-streptomycin (100 μg/ml) and 10% (v/v) FBS at 37 °C in a humidified atmosphere of 5% CO_2_.

### MTT assay and LDH assay

The MTT assay was conducted for cell viability investigation as previously described^46^. A2780/WT cells and A2780/PTX^R^ cells were seeded in 96-well plates at a final concentration of 5 ×10^3^ cells/well. After a 24-hour incubation for adhesion, cells were treated with EVO at a series of concentrations. After a 24-hour incubation, cell viability was incubated with MTT solution (1 mg/ml) for four hours. The formazan crystal formation was dissolved with DMSO and determined by absorbance at 570 nm using a micro-plate reader (SpectraMax M5, Molecular Devices). Cell viability was expressed as a percentage of the vehicle control. The LDH release from cells after EVO treatment was determined with a commercial kit according to the manufacturers’ protocol (Roche). The percentage of LDH release was calculated as per our previous reports^47^.

### Morphology observation

A2780/WT and A2780/PTX^R^ cells were exposed to different doses of EVO for 24 hours. After the indicated treatments, cell morphology was observed and captured using a microscope (Olympus MVX10, Japan) equipped with a digital camera (ColorView II, Soft Imaging System, Olympus), to survey cell counts and morphology alteration at 100 × magnification. The representative images were based on at least three independent experiments.

### Colony formation assay

Cells were plated in 6-well plates with 5 × 10^2^ cells per well in duplicate. After a 15-day incubation at 37 °C for visible colonies, these colonies were fixed with 4% paraformaldehyde for 15 minutes and stained with crystal violet for five minutes. The number of colonies (≥50 cells as a colony) was captured with a microscope (Olympus MVX10, Japan) equipped with a digital camera (ColorView II, Soft Imaging System, Olympus).

### CFDA-SE cell proliferation assay

Cell proliferation determination was conducted by CFDA-SE probe. Briefly, cells (5 × 10^2^) were seeded and stained with CFDA-SE in 6-well plates according to the manufacturer’s protocol. Then, cells were exposed to a series of concentrations of EVO for six days. CFDA-SE fluorescence was detected by flow cytometry (BD FACS Canto™, BD Biosciences, San Jose, USA) and calculated by FlowJo software (Treestar, Ashland, OR, USA).

### Cell cycle assay

Cell cycle distribution was determined as previously described^48^. After the indicated treatments, cells were washed with cold PBS and harvested by centrifugation. Then, cells were re-suspended in 70% (v/v) cold ethanol and stored at −20 °C overnight. After 30-minute incubation with propidium iodide (PI) solution in the dark, cell cycle distribution was analyzed by flow cytometry (BD FACS Canto™, BD Biosciences, San Jose, USA). Results were calculated by Mod Fit LT software (version 3.0).

### Western blotting

A2780/WT and A2780/PTX^R^ cells were treated with different concentrations of EVO for 24 hours, and the total protein was extracted with RIPA lysis buffer containing 1% phenylmethane- sulfonylfluoride (PMSF) and 1% protease inhibitor cocktail. As per our previous report^48^, the BCA protein assay kit (Pierce) was applied to determine protein concentrations. Equal amounts of total protein were subjected to sodium dodecyl sulfate-polyacrylamide gel electrophoresis (SDS-PAGE) and were transferred onto a polyvinylidene fluoride (PVDF) membrane. Blocking overnight was at 4 °C with 5% non-fat milk. The membrane was incubated for two hours with the primary antibodies (dilution ration 1:1000) at room temperature, including Cyclin B1, p27, p21, Rb, p-Cdc2 (Thr161), Cdc2, p-ERK (Thr202/Tyr204), ERK, p38, Ras, GAPDH and β-actin, and the incubation for the secondary antibodies was one hour at room temperature. Bands visualization was conducted by an ECL Advance Western Blotting Detection Kit (Amersham, UK). The densities were calculated by the Quantity One Software (Bio-Rad, CA, USA) and were normalized by β-actin.

### Dual-luciferase reporter assay

A2780/PTX^R^ cells (1 × 10^4^) were plated per well in a 24-well plate overnight. A2780/PTX^R^ cells were co-transfected with 0.8 μg pRb-luc and 0.8 μg pRL-TK as a transfection efficiency control. The plasmids and TurbotFect transfection reagent were diluted in Opti-MEM reduced serum medium according to TurbotFect transfection reagent protocol. The diluted DNA was mixed together with diluted TurbotFect transfection reagent at a 1:2 ratio and incubated at 25 °C for 20 minutes. 100 μL of complexes was transferred to each well. After overnight incubation, the cells were refreshed and cultured in the completed medium for an additional 24 hours. Then, cells were exposed to a series of concentrations of EVO for 24 hours. Cell lysates were collected by passive lysis buffer, and were detected using the SpectraMax M5 microplate reader. Resulting data were normalized to pRL-TK values.

### siRNA-mediated RNA interference

The detailed procedure for performing target gene silencing has been described previously^49^. In our study, we used the Cyclin B1 siRNA: 5′-CCAAACCUUUGUAGUGAAUTT-3′ (Seq. I). We also used another siRNA sequence, 5′-GGUUGUUGCAGGAGACCAUTT -3′ (Seq. II), for silencing the Cyclin B1 gene and then investigated the Cyclin B1 expressions and cell cycle distribution. A FAM siRNA duplex with the target sequence, 5′-CGGCAAGCUGACCCUGAAGTT-3′ was employed as a non-silencing control. A2780/PTX^R^ cells were transfected with siRNAs using Lipofectamine 2000 according to the manufacturer’s instructions (Invitrogen, Carlsbad, CA, USA). After a 4-hour transfection, the cells were cultivated in the completed medium for an additional 48 hours. Cyclin B1 siRNA-transfected or control A2780/PTX^R^ cells (2 × 10^5^) were co-cultured in 6-well plates. After 24 hours of EVO treatments (10 μM), cell cycle distribution was analyzed by flow cytometry (BD FACS Canto™, BD Biosciences, San Jose, USA). Results were calculated by Mod Fit LT software (version 3.0).

### P-gp expression assay

P-gp expression was evaluated by the antibody P-glycoprotein conjugated FITC (BD Biosciences). A2780/WT and A2780/PTX^R^ cells were seeded onto 6-well plates, and the cells were treated with different concentrations of EVO for 24 hours. Next, cells were harvested and incubated with 100 μl P-gp antibody dye-loading buffer at 37 °C for 30 minutes protected from light. The FITC fluorescence was detected using flow cytometry (BD FACS Canto™, BD Biosciences, San Jose, USA). All experiments were performed in triplicate and compared to negative controls.

### P-gp function assay

Calcium AM was used to determine the activity of P-gp. A2780/PTX^R.^ cells were seeded onto 6-well plates, and the cells were treated with different concentrations of EVO for one hour. Next, 100 μl calcium AM dye-loading solution was added to each well and incubated at 37 °C for 30 minutes protected from light. Cells were harvested and intracellular fluorescence was detected using flow cytometry (BD FACS Canto™, BD Biosciences, San Jose, USA). All experiments were performed in triplicate and compared to negative controls.

### Statistical Analysis

All data represent the mean of at least three separately performed experiments. The significance of variations was evaluated by GraphPad Prism software (GraphPad Software, USA). Student’s *t*-test was used for statistical comparison. P values less than 0.05 was considered significant.

## Results

### The effect of EVO on cell viability of human epithelial ovarian cancer cells A2780/WT, A2780/PTX^R^, and normal cell RAW 264.7

**Figure 1:**
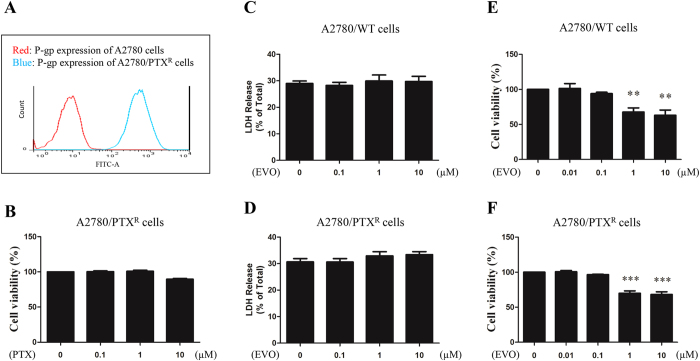
The effect of EVO on cell viability and LDH release of human epithelial ovarian cancer cells A2780/WT and A2780/PTX^R^. **(A)** Human epithelial ovarian cancer cells A2780/WT and A2780/PTX^R^ were assessed by P-gp protein expression using the FITC-P-gp antibody. **(B)** Cell viability of A2780/PTX^R^ cells was detected after PTX treatments (24 hours). The LDH release from A2780/WT **(C)** and A2780/PTX cells **(D)** was determined by LDH assay after EVO treatments (24 hours, 0, 0.1, 1 and 10 μM); also the cell viability of A2780/WT **(E)** and A2780/PTX^R^ **(F)** cells was tested by MTT assay. LDH release values were represented as percentage of the total and cell viability ratios were represented as percentage as the vehicle control. Data were expressed as mean ± SE of three independent experiments. **P < 0.01 and ***P < 0.001.

Paclitaxel-sensitive and -resistant A2780 cells were obtained and assessed by P-gp protein expression. As shown in Fig. 1A, A2780/PTX^R^ cells were differentiated by florescence intensity from A2780/WT cells as a result of the elevated P-gp protein. Viability of the A2780/PTX^R^ cells after PTX treatments (0.1, 1 and 10 μM) for 24 hours was assessed by MTT assay, and no significant variations were observed in Fig. 1B. Those results showed that A2780/PTX^R^ cells were resistant to paclitaxel, with an IC_50_ value of 550.9 μM.

As shown in Fig. 1C,D, the cytotoxicity induced by EVO in A2780/WT and A2780/PTX^R^ cells was determined by the LDH assay. After a series of EVO (0.1, 1 and 10 μM) treatment for 24 hours, no significant variations were observed in both cell lines. Then, the cell viability after EVO treatment (0.01, 0.1, 1, 10 μM) for 24 hours in both cell lines was assessed by the MTT assay. As shown in Fig. 1E,F, 1 μM and 10 μM of EVO significantly inhibited cell viability in both cell lines. Otherwise, EVO (0 to 10 μM) exhibited no effect on RAW 264.7 cell viability after 24 hours treatment, with an IC_50_ value of 132.1 μM (Fig. s1A).

### The effect of EVO on malignant proliferation of A2780/WT and A2780/PTX^R^ cells

**Figure 2:**
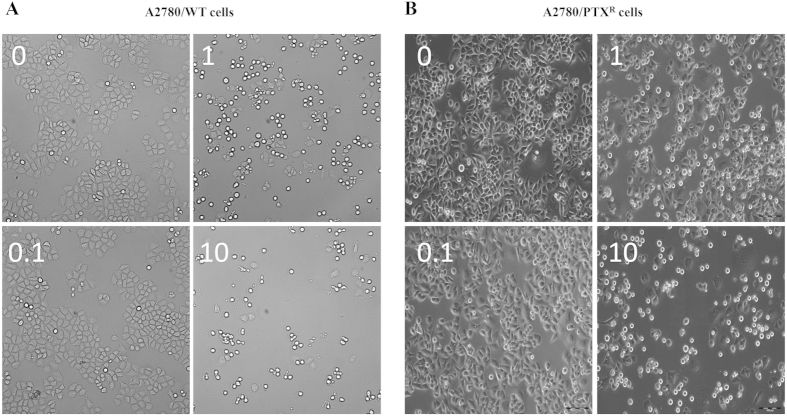
Morphology alterations of A2780/WT and A2780/PTX^R^ cells. Human epithelial ovarian cancer cells A2780/WT (**A**) and A2780/PTX^R^ (**B**) were observed by microscopy. The morphology alteration and cell number were visualized after EVO treatments (24 hours at 0, 0.1, 1 and 10 μM).

**Figure 3:**
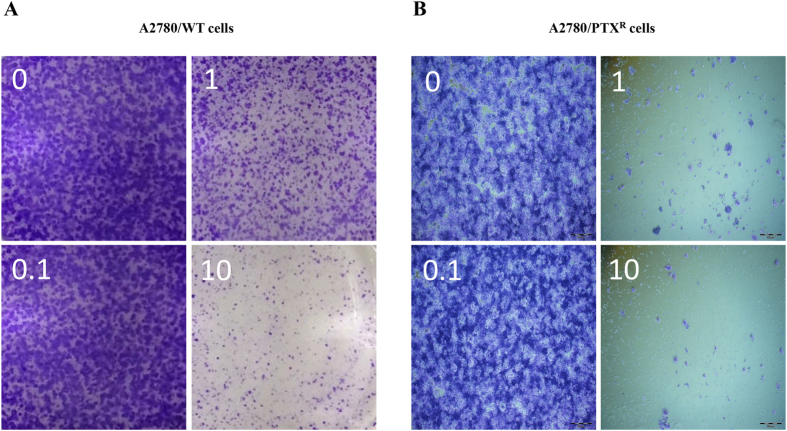
Colony formation alterations of A2780/WT and A2780/PTX^R^ cells. Human epithelial ovarian cancer cells A2780/WT (**A**) and A2780/PTX^R^ (**B**) were determined by the colony formation assay, and the colony formation status was visualized by microscopy after EVO treatments (15 days at 0, 0.1, 1 and 10 μM).

**Figure 4:**
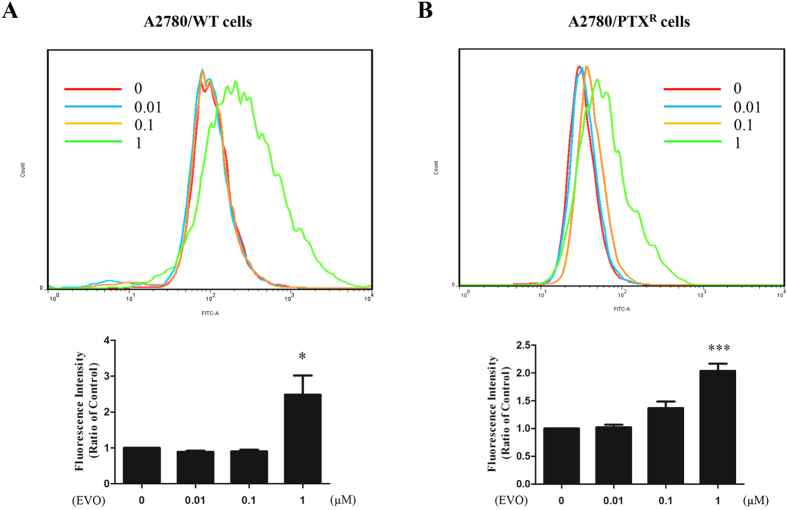
The effect of EVO on malignant proliferation of human epithelial ovarian cancer cells A2780/WT and A2780/PTX^R^. The proliferation of human epithelial ovarian cancer cells, A2780/WT **(A)** and A2780/PTX^R^ (**B**) was determined by the CDFA-SE assay, and the malignant proliferation was calculated by fluorescence alterations after EVO treatments (6 days at 0, 0.01, 0.1 and 1 μM). The alterations of fluorescence intensity were represented as percentages of the vehicle control. Data were expressed as mean ± SE of three independent experiments. *P < 0.05 and ***P < 0.001.

After confirming a significant variation of cell viability induced by EVO, morphology observation was conducted to visualize the influence on the proliferation of A2780/WT and A2780/PTX^R^ cells. At concentrations of 1 μM and 10 μM, the cell population showed dramatic depletions after EVO incubation in both cell lines, with a reduction exceeding 50 percentages as shown in Fig. 2A,B. Further, in the clone formation assay, steep declines were observed after 1 μM and 10 μM EVO treatment in A2780/WT cells, as shown in Fig 3A, and in Fig. 3B, almost no clones were observed after 1 μM and 10 μM EVO treatment in A2780/PTX^R^ cells. The proliferation influence was also assessed by CFDA-SE assay through fluorescence alterations. In Fig. 4, after a series of EVO (0.01, 0.1 and 1 μM) treatment for six days, cell proliferation was assessed, and 1 μM of EVO significantly suppressed cell proliferation in A2780/WT and A2780/PTX^R^ cells. Proliferation suppression was found to be much more obvious in A2780/PTX^R^ cells with a gradual increase, as shown in the colony formation assay.

### EVO induced cell cycle arrest in dose- and time-dependent manners

**Figure 5:**
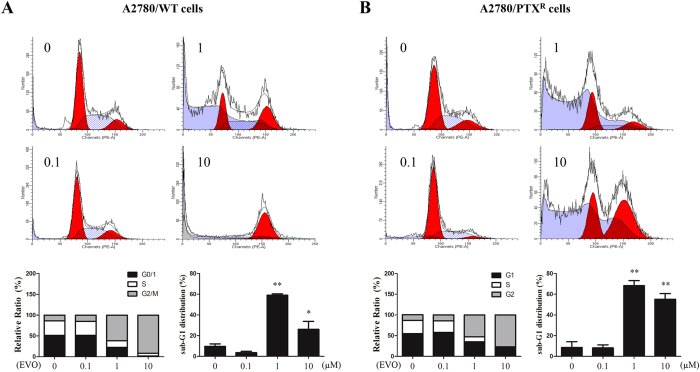
EVO induced cell cycle arrest in a dose-dependent manner. Cell cycle distributions of A2780/WT **(A)** and A2780/PTX^R^ (**B**) were conducted by flow cytometer analysis after EVO treatments (24 hours at 0, 0.1, 1 and 10 μM). Histograms represented cell cycle distribution and the ratios were calculated. *P < 0.05 and **P < 0.01.

**Figure 6:**
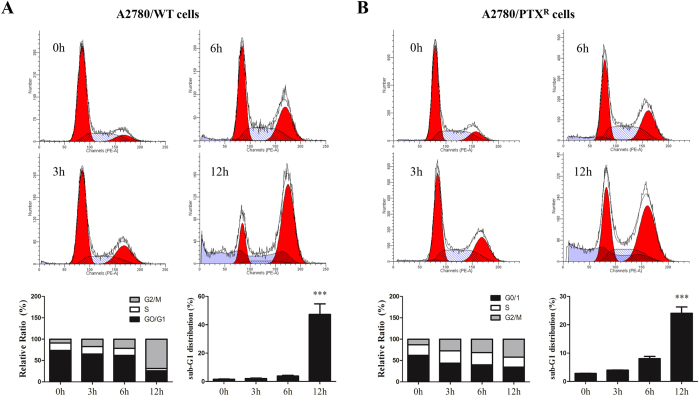
EVO induced cell cycle arrest in a time-dependent manner. Cell cycle distributions of A2780/WT (**A**) and A2780/PTX^R^ (**B**) were conducted by flow cytometer analysis after EVO treatment (1 μM at 0, 3, 6 and 12 hours). Histograms represented cell cycle distribution and the ratios were calculated. ***P < 0.001.

Cell cycle distribution was conducted by flow cytometer analysis, and a series of concentrations of EVO (0.1, 1 and 10 μM) induced G2/M phase arrest with a significance observed in A2780/WT and A2780/PTX^R^ cells, as shown in Fig. 5. In the time courses, G2/M phase arrest was also found after different durations (0, 3, 6, and 12 hours), as shown in Fig. 6. Therefore, EVO was confirmed to induce G2/M phase arrest in a dose- and time-dependent manner in both cell lines. Moreover, EVO also significantly induced sub-G1 phase arrest in both cell lines. Those results indicated that EVO promoted apoptotic death in ovarian cancer cells.

### The underlying mechanism of the anti-proliferative effect of EVO

**Figure 7:**
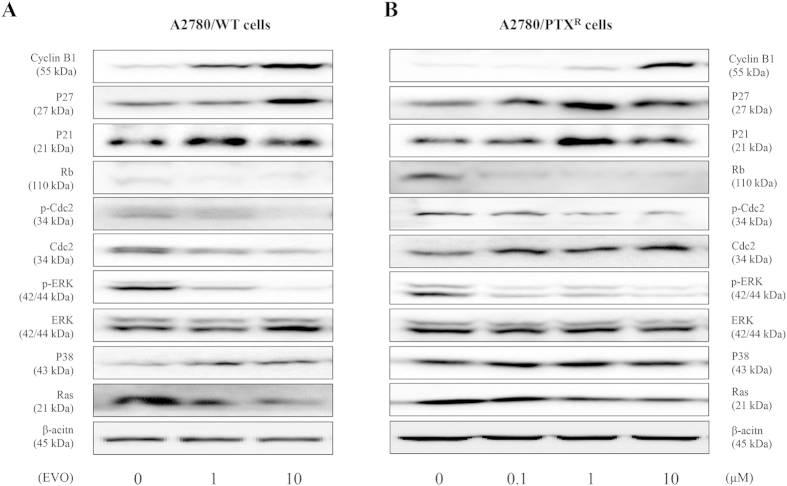
The underlying mechanisms of anti-proliferative effect of EVO on human epithelial ovarian cancer cells, A2780/WT and A2780/PTX^R^. The involved proteins of A2780/WT (**A**) and A2780/PTX^R^ (**B**) were detected by western blotting after EVO treatments (24 hours).

After the EVO treatment mentioned above, the expression alterations of related proteins in A2780/WT and A2780/PTX^R^ cells were determined by western blotting, as shown in Fig. 7. EVO up-regulated Cyclin B1, p27, and p21 in both cell lines, down-regulated Rb, and inhibited Cdc2 (Thr161) phosphorylation and ERK (Thr202/Tyr204) phosphorylation in both cell lines. Furthermore, EVO decreased Ras protein and increased p38 protein expression in A2780/WT and A2780/PTX^R^ cells. Additionally, EVO down-regulated Cdc2 expression in A2780/WT cells, but up-regulated Cdc2 expression in A2780/PTX^R^ cells. We further investigated other alterations in CDK induced by EVO in A2780/PTX^R^ cells. Our results showed that EVO continued to up-regulate CDK4 expression, and down-regulate the protein expressions of CDK2 and CDK6 in A2780/PTX^R^ cells (Fig. s1B).

**Figure 8:**
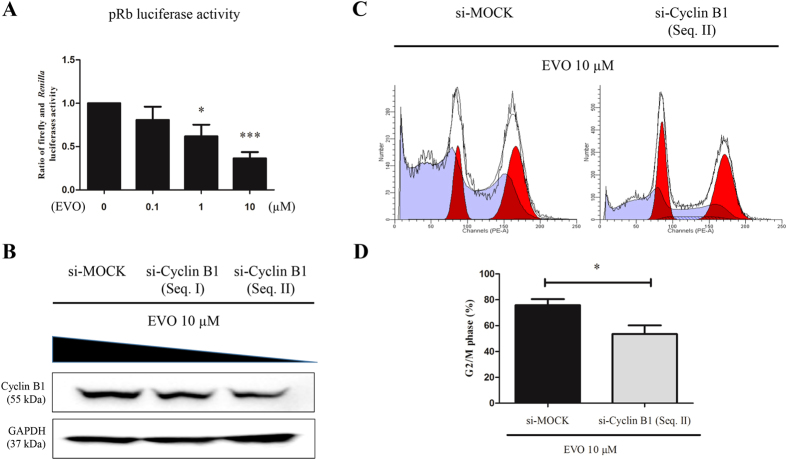
EVO induced G2/M phase cell cycle arrest involvement of pRb activation and Cyclin B1 regulation in A2780/PTX^R^ cells. (**A**) A2780/PTX^R^ cells transfected with pRb luciferase reporter gene were treated with 0, 0.1, 1 and 10 μM of EVO for 24 hours, and pRb-driven transcription activity was detected by using the dual-luciferase reporter system. (**B**) A2780/PTX^R^ cells were treated with 10 μM EVO after transfection with siRNAs using Lipofectamine 2000, and Cyclin B1 expression was measured by western blotting. (**C**) Knocking-down Cyclin B1 could attenuate the G2/M phase arrest induced by EVO (10 μM) in A2780/PTX^R^ cells. (**D**) Histograms represented cell cycle distribution at G2/M phase, and the ratios were calculated. Data were expressed as mean ± SE of three independent experiments. *P < 0.05 and ***P < 0.001.

Next, we illustrated the effect of EVO on pRb transcriptional activity in A2780/PTX^R^ cells. Cells were transiently co-transfected with pRb-luc and pRL-TK, and then treated with a series of concentrations of EVO (0.1, 1 and 10 μM). Our results showed that EVO significantly decreased pRb transcriptional activity in a dose-dependent manner (Fig. 8A). In our pre-experiments, western blotting assay confirmed that the knock-down effect of si-Cyclin B1 (Seq. II) was stronger than si-Cyclin B1 (Seq. I) (Fig. s1C and D). As a result, Cyclin B1 siRNA-transfected or control A2780/PTX^R^ cells were co-cultured for our next experiment in 6-well plates where the high transfection efficacy was also observed (Fig. s1E). Overall, EVO was found to have different effects on multiple transfected cells (Fig. 8B) including the observation that knocking-down Cyclin B1 could attenuate the G2/M phase arrest that is induced by EVO (10 μM) in A2780/PTX^R^ cells (Fig. 8C,D).

**Figure 9:**
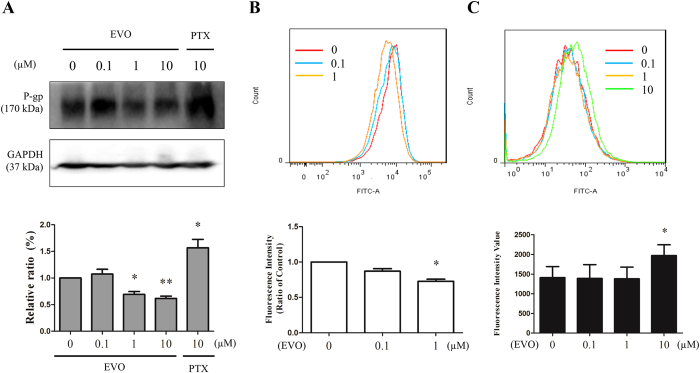
EVO improved chemo-resistance partly due to involvement of MDR-1 in A2780/PTX^R^ cells. **(A)** A2780/PTX^R^ cells were treated with 0, 0.1, 1 or 10 μM of EVO for 24 hours, and the expression level of MDR-1 was detected by western blotting and calculated as a percentage of vehicle control. (**B**) The expression alterations of MDR-1 were confirmed by FITC-P-gp antibody staining using flow cytometry after EVO treatments (24 hours) and were represented as a percentage of the vehicle control. (**C**) P-gp function evaluation was assessed by calcium AM incubation (30 minutes) using flow cytometry after EVO treatments (1 hour). Data were expressed as mean ± SE of three independent experiments. *P < 0.05 and **P < 0.01.

In A2780/PTX^R^ cells, 1 μM and 10 μM of EVO dramatically decreased the expression level of MDR-1 evaluated by western blotting, as shown in Fig. 9A. Meanwhile, FITC-P-gp staining assay by flow cytometry showed that EVO also inhibited MDR-1 protein expression at a concentration of 1μM in A2780/PTX^R^ cells, as shown in Fig. 9B. In the calcium AM staining assay, P-gp function evaluation was assessed further by flow cytometry, as shown in Fig. 9C. After one hour pro-treatment of EVO, 30-minute incubation with calcium AM was conducted to determine fluorescence alterations. 10 μM of EVO induced a fluorescence enhancement of calcium AM, also indicating the inhibition of P-gp function in A2780/PTX^R^ cells.

## Discussion

In our present study, human epithelial ovarian cancer cells, A2780 were utilized to establish a chemo-resistant cell line through stepwise concentration exposure, in order to investigate the chemo-resistance influence of an active constituent from Chinese medicine. The resistance to PTX was also confirmed by a lack of obvious variation of cell viability through the MTT assay in A2780/PTX^R^ cells. The effect of EVO on cell viability of human epithelial ovarian cancer cells, A2780/WT and A2780/PTX^R^ was conducted through the MTT assay. Remarkable inhibitions on cell viability such as the high concentrations (1 μM and 10 μM) were much more pronounced than the low concentrations (0.01 μM and 0.1 μM) and were observed in both A2780/WT and A2780/PTX^R^ cells. Notably, this is the first time that the effects of EVO on human epithelial ovarian cancer and resistant human epithelial ovarian cancer have been investigated. Although there have been intensive reports on a variety of cancer types, this is the first time that the proliferation inhibition in epithelial ovarian cancer cells, A2780/WT and the related chemo-resistant cell A2780/PTX^R^ has been confirmed.

In this experiment, no significant alterations of the LDH release levels have been observed which would indicate that EVO induced cell viability inhibition but caused no cytotoxic effect. EVO also exhibited no cytotoxic effect on RAW 264.7 cells even at 10 μM. These findings suggested that EVO maintains certain selectivity to cancer cells. To verify, the morphology of the cell number change was visualized, and representative images of colony formation also confirmed the decreased growth rates of both cell lines. The proliferation influence of EVO was also confirmed by CFDA-SE assay through fluorescence alterations. Overall, the potential anti-proliferative effect of EVO might be induced through cell cycle arrest at the G2/M phase and the sub-G1 phase.

The cyclin-dependent kinases (CDKs), specifically CDK1, CDK2, CDK4, and CDK6 play an important role in regulating cell cycle^50^. The activation of CDK1 (Cdc2), which is close to G2/M phase cell cycle arrest, promotes mitosis in cell cycle progression, which requires cyclin binding and phosphosrylation activation at Thr161. Meanwhile, EVO inhibits Cdc2 phosphorylation at Thr161. Therefore, the activation failure of Cdc2 still occupies the main position after EVO treatment. This activation failure might be consistent with the induced cell cycle arrest at the G2/M phase. Moreover, EVO down-regulated Cdc2 expression in A2780/WT cells, but up-regulated Cdc2 expression in A2780/PTX^R^ cells. It has been reported that inhibition of certain cell cycle CDKs may be compensated for by other CDKs^51^, thus additional CDKs alterations induced by EVO were further investigated in A2780/PTX^R^ cells. Our results suggested that EVO inhibition on the protein expressions of CDK2 and CDK6 could be compensated for by Cdc2 and CDK4 in A2780/PTX^R^ cells.

In our present study, the increase of Cyclin B1 induced by EVO was observed robustly in both A2780/WT and A2780/PTX^R^ cells. This was also the first time that EVO increased the expression level of Cyclin B1 in human epithelial ovarian cancer, A2780 and the related PTX-resistant cell lines. Although Cyclin B1 was always over-expressed and led to uncontrolled growth in many cancer cell lines, when in reference to human ovarian cancer cells, there were a few controversial reports. For instance, besides the suppression of Cyclin B1 expression^52^,^53^,^54^,^55^, there was also a Cyclin B1 increase in correspondence with the S-phase arrest induced by taxol and cisplatin in cisplatin-resistant A2780 cells^56^. There was also an up-regulation of Cyclin B1 in ovarian cancer cells, A2780-1A9 that was observed while overcoming multidrug resistance^57^. Therefore, the activation failure of Cdc2 might be attributed to an overall expression of other unknown activating factors and might require further investigation.

p27 and p21 are regarded as tumor suppressors involved in regulating cell cycle progression. The up-regulated expressions of p27 and p21 induced by EVO in both A2780/WT and A2780/PTX^R^ cells also indicate activation failure of Cdc2. The inactivation of Rb cooperated with cyclin-dependent kinase activation in order to promote cell division and proliferation. Although Rb suppression by EVO might likely contribute to cell cycle progression, the activation failure or inhibition of Cdc2 mentioned above would also determine the eventual commitment of cell cycle arrest. Actually, reports showed that tumor development was regulated by the cooperation of p27 and Rb through integrating regulatory signals. As the main factors in the MAPK pathway, ERK1/2 phosphorylation and p38 expression showed different responses after EVO treatments, remarkable inhibition and increased alteration, respectively. The significant inhibition of ERK1/2 phosphorylation was consistent with the anti-proliferative effect of EVO. The over-expressed Ras was closely related to the MAPK signaling pathway, and the decreased level induced by EVO indicated an enhancing anti-proliferative potential in human epithelial ovarian cancer, A2780 and the related PTX-resistant cell lines. Besides, the expression level of MDR-1 was reduced after EVO treatment in A2780/PTX^R^ cells. Those results were confirmed by both western blotting and flow cytometry. A fluorescence enhancement of calcium AM gave further testimony to MDR-1 function inhibition by EVO in chemo-resistance.

In our present study, EVO was observed to have significant proliferation suppression for the first time in human epithelial ovarian cancer cells, A2780 and the related PTX-resistant cell line. The declined clone formation and representative fluorescence of CFDA-SE both confirmed this inhibition of malignant proliferation. Meanwhile, EVO induced cell cycle arrest in a dose- and time-dependent manner, as confirmed by FACs assay. This cell division disturbance might be mediated by the cooperation of Cyclin B1 and Cdc2, including the up-regulation of Cyclin B1, p27, p21, and activation failure of Cdc2. The regulation of the MAPK signaling pathway also assisted in this process. Furthermore, EVO significantly decreased pRb transcriptional activity, and knocking-down Cyclin B1 could attenuate the G2/M phase arrest induced by EVO (10 μM) in A2780/PTX^R^ cells. Our results indicated that EVO suppressed cell proliferation through G2/M phase cell cycle arrest via regulation of the Rb and Cyclin B1 signaling pathways. EVO also indicated an enhancing chemo-sensitivity potential in A2780/PTX^R^ cells, through down-regulating the expression level of MDR-1 accompanied with MDR-1 function suppression.

Taken together, we confirmed that EVO exerted an anti-proliferative effect on human epithelial ovarian cancer cells and resistant human epithelial ovarian cancer cells, through inducing cell cycle arrest at the G2/M phase. It was also involved in Cyclin B1/Cdc2, p27/Rb, MAPK signaling pathways, and it improved chemo-resistance partly by contributing to the suppression of P-gp protein expression and function. Overall, we found that EVO significantly suppressed malignant proliferation in human epithelial ovarian cancer, thus proving to be a potential anti-cancer agent in the future.

## Additional Information

**How to cite this article**: Zhong, Z.-F. *et al.* Anti-proliferative activity and cell cycle arrest induced by evodiamine on paclitaxel-sensitive and -resistant human ovarian cancer cells. *Sci. Rep.* **5**, 16415; doi: 10.1038/srep16415 (2015).

## Supplementary Material

Supplementary Information
