# Supplementary material for: Anti-proliferative activity and cell cycle arrest induced by evodiamine on paclitaxel-sensitive and -resistant human ovarian cancer cells
Source: Sci Rep. 2015 Nov 10;5:16415. doi: 10.1038/srep16415 (PMC4639765; doi:10.1038/srep16415)
Supplement: Supplementary Information [file srep16415-s1.pdf]

Supporting Information

Anti-proliferative activity and cell cycle arrest induced by evodiamine on paclitaxel-sensitive and -resistant human ovarian cancer cells

Zhang-Feng Zhong[1], Wen Tan[3,*], Sheng-Peng Wang[1], Wen-An Qiang[2,#], Yi-Tao Wang[1,#]

1. University of Macau, Institute of Chinese Medical Sciences, State Key Laboratory of Quality Research in Chinese Medicine, Macau, China

2. Division of Reproductive Biology Research, Department of Obstetrics and Gynecology, Feinberg School of Medicine at Northwestern University, Chicago, Illinois, United States of America

3. School of Pharmacy, Lanzhou University, Lanzhou, Gansu, China

MATERIALS AND METHODS

Materials and reagents

EVO and 3-[4,5-dimethyl-2-thiazolyl]-2,5-diphenyl tetrazolium bromide (MTT) were purchased from Sigma-Aldrich (St. Louis, MO). Dulbecco's Modified Eagle Medium (DMEM), fetal bovine serum (FBS), penicillin (100 U/ml)-streptomycin (100 μg/ml), phosphate-buffered saline (PBS) and 0.25% w/v trypsin/1 mM EDTA from Gibco Life Technologies (Grand Island, USA) were used for cell culture. The primary and secondary antibodies against Cyclin B1, CDK2, CDK4, CDK6, GAPDH, and β-actin

were purchased from Cell Signaling Technology (Danvers, MA).

Cell culture

RAW 264.7 cells were employed as normal cells to identify the selective effect of EVO. The paclitaxel-resistant A2780 cells were established by stepwise exposure to increased concentrations of paclitaxel, as usually described [1]. Cells were cultured in RPMI 1640 medium with penicillin (100 U/ml)-streptomycin (100 μg/ml) and 10% (v/v) FBS at 37 °C in a humidified atmosphere of 5% CO_2.

MTT assay

The MTT assay was conducted for cell viability investigation as previously described [2]. RAW 264.7 cells (5×10^3) were seeded in 96-well plates at a final concentration of 5×10^3 cells/well. After a 24-hour incubation for adhesion, cells were treated with EVO at a series of concentrations. After a 24-hour incubation, cell viability was incubated with MTT solution (1 mg/ml) for four hours. The formazan crystal formation was dissolved with DMSO and determined by absorbance at 570 nm using a micro-plate reader (SpectraMax M5, Molecular Devices). Cell viability was expressed as a percentage of the vehicle control.

Western blotting

A2780/PTX^R cells were treated with different concentrations of EVO for 24 hours, and the total protein was extracted with RIPA lysis buffer containing 1%

phenylmethane- sulfonylfluoride (PMSF) and 1% protease inhibitor cocktail. As per our previous report [3], BCA protein assay kit (Pierce) was applied to determine protein concentrations. Equal amounts of total protein were subjected to sodium dodecyl sulfate-polyacrylamide gel electrophoresis (SDS-PAGE) and were transferred onto a polyvinylidene fluoride (PVDF) membrane. Blocking overnight was at 4 °C with 5% non-fat milk. The membrane was incubated for two hours with the primary antibodies (dilution ration 1:1000) at room temperature, including Cyclin B1, CDK2, CDK4, CDK6, GAPDH and β-actin, and the secondary antibodies incubation was one hour at room temperature. Band visualization was conducted by an ECL Advance Western Blotting Detection Kit (Amersham, UK). The densities were calculated by the Quantity One Software (Bio-Rad, CA, USA) and were normalized by β-actin or GAPDH.

siRNA-mediated RNA interference

The detailed performing for a target gene silencing has been described previously [4]. In our study, we used the Cyclin B1 siRNA: 5'-CCAAACCUUUGUAGUGAAUTT-3' (Seq. I). We also used another siRNA sequence, 5'-GGUUGUUGCAGGAGACCAUTT -3' (Seq. II), for silencing the Cyclin B1 gene and then investigated the Cyclin B1 expressions and cell cycle distribution. A FAM siRNA duplex with the target sequence 5'-CGGCAAGCUGACCCUGAAGTT-3' was employed as a non-silencing control. The other non-silencing control was Control siRNA (FITC Conjugate) purchased from Santa Cruz (Santa Cruz, USA). A2780/PTX^R cells were transfected with siRNAs using Lipofectamine 2000 according to the manufacturer's instructions (Invitrogen, Carlsbad, CA, USA). After a

4-hour transfection, the cells were cultivated in the completed medium for an additonal 48 hours. Cyclin B1 siRNA-transfected or control A2780/PTX^R cells (2 × 10^5) were co-cultured in 6-well plates.

Statistical Analysis

All data represent the mean of at least three separately performed experiments. The significance of variations was evaluated by GraphPad Prism software (GraphPad Software, USA). P values less than 0.05 was considered significant.

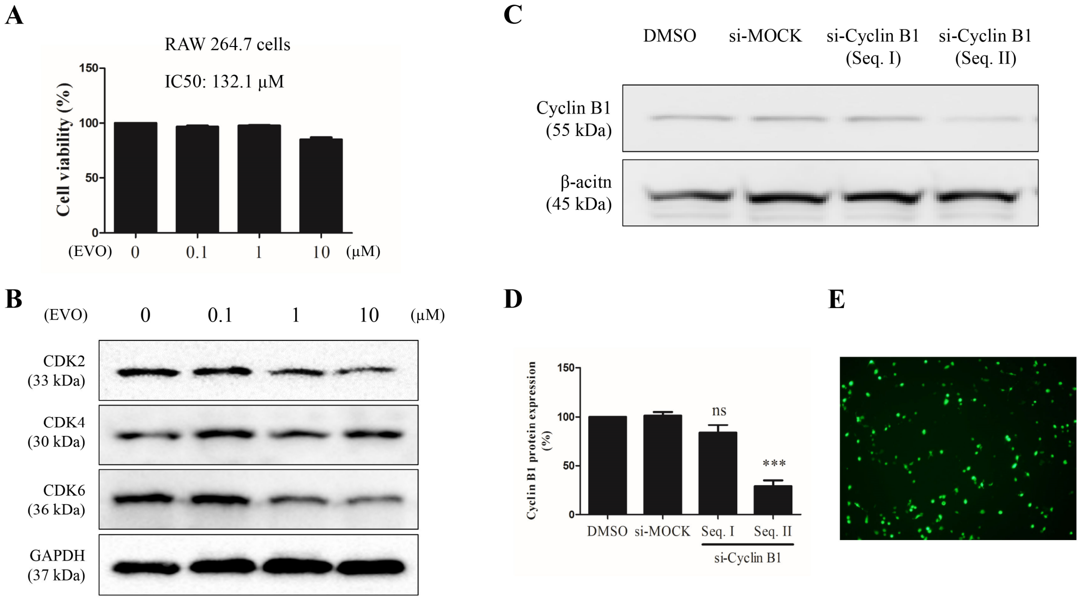

Figure s1. The effect of EVO on cell viability in RAW 264.7 cells, on protein expressions in A2780/PTX^R cells, and the transfection efficacy of siRNAs. (A) Effect of EVO on the cell viability of RAW 264.7 cells was tested by the MTT assay. **(B)** The involved proteins of A2780/PTX^R were detected by western blotting after EVO treatment (24 hours at 0, 0.1,1 and 10 μM). **(C)** A2780/PTX^R cells were

transfected with siRNAs using Lipofectamine 2000, and Cyclin B1 expression was measured by western blotting. **(D)** Histograms represented Cyclin B1 protein expression and the ratios were calculated. **(E)** A2780/PTX^R cells were transfected with Control siRNA (FITC Conjugate) using Lipofectamine 2000, photographs were taken at ×100 magnifications using a digital camera. Data were expressed as mean ±SE of three independent experiments. ns stands for not significant and *** $P <$ 0.001.

REFERENCES

1. Li, Z. *et al.* MiR-27a modulates MDR1/P-glycoprotein expression by targeting HIPK2 in human ovarian cancer cells. *Gynecologic oncology* **119**, 125-130 (2010).
2. Zahedifard, M. *et al.* Synthesis, characterization and apoptotic activity of quinazolinone Schiff base derivatives toward MCF-7 cells via intrinsic and extrinsic apoptosis pathways. *Scientific Reports* **5**, 11544 (2015).
3. Zhong, Z., Tan, W., Chen, X. & Wang, Y. Furanodiene, a natural small molecule suppresses metastatic breast cancer cell migration and invasion *in vitro*. *European journal of pharmacology* **737**, 1-10 (2014).
4. Liang, L. *et al.* Dihydroquercetin (DHQ) induced HO-1 and NQO1 expression against oxidative stress through the Nrf2-dependent antioxidant pathway. *Journal of agricultural and food chemistry* **61**, 2755-2761 (2013).
